# Performance of immunochromatographic and immunoenzymatic techniques in the diagnosis of toxoplasmosis in pregnant women in Cameroon: need for harmonization

**DOI:** 10.11604/pamj.2022.43.195.36996

**Published:** 2022-12-16

**Authors:** Jacqueline Félicité Yéngué, Claudine Mireille Ntsama Essomba, Georgia Elna Ambada Ndzengue, Nelson Sonela, Elise Elong Lobè, Michel Carlos Tommo Tchouaket, Aude Christelle Ka’e, Jules Colince Tchadji, Sebastien Kenmoe, Aubin Joseph Nanfack, Charles Félix Bilong Bilong

**Affiliations:** 1 Department of Animal Biology and Physiology, University of Yaoundé I, Yaoundé, Cameroon,; 2 Department of Immunology, Chantal Biya International Reference Center for Research on HIV/AIDS Prevention and Management (CIRCB), Yaoundé, Cameroon,; 3 Department Center for Pharmaceutical Biotechnology, University of Tor Vergata, Roma, Italy,; 4 Department of Bacteriology and Virology, Catholic University of Central Africa, Yaoundé, Cameroon,; 5 Department of Microbiology and Parasitology, University of Buea, Buea Cameroon

**Keywords:** Toxoplasmosis, pregnant women, diagnosis, RDT, ELISA, performance, Cameroon

## Abstract

**Introduction:**

in order to contribute to the improvement of the management of toxoplasmosis in pregnant women in Cameroon, performance of two techniques commonly used in the diagnosis of toxoplasmosis was evaluated.

**Methods:**

a total of 541 pregnant women were recruited from seven hospitals in two Regions of Cameroon, of which 63% (341: Batch1) were from health facilities (HF) using a immunochromatographic technique (ICT) as a screening test for toxoplasmosis, and 37% (200: Batch2) from those using an immunoenzymatic technique (IEZ). On each sample, Ig (Immunoglobulin) G (IgG) and IgM were tested by three techniques: a Rapid Diagnostic Test (RDT), an Enzyme Linked Immuno Sorbent Assay (ELISA) and a Vidas Enzyme-linked fluorescent assay taken as reference (VIDAS/ELFA). The results from the health facilities were recorded.

**Results:**

for the IgG assay, our two laboratory methods were sensitive (96.0% and 97.5%) and specific (64.2% and 59.7%). Their concordance rates with the VIDAS/ELFA reference were above 60% (P<0.001). Moreover, for the IgM assay, the performances of the two methods were equivalent: Se= 18.2%, Sp= 99.4% with a low concordance rate (Kappa = 0.24). Considering the results provided by the selected hospitals, the ELISA used in Batch2 showed similar performances to the two techniques used in reference lab while the performances were low for the RDT used in Batch1.

**Conclusion:**

both methods showed similar performances (good for (IgG) and poor for IgM). However, for the immunochromatographic method, differences in performance were found between our results and those provided by the selected health facilities. These differences suggest a harmonization of diagnostic techniques for toxoplasmosis in pregnant women in Cameroonian health facilities.

## Introduction

Toxoplasmosis, anthropozoonosis, remains a public health problem. It is a cosmopolitan infectious disease, affecting about one third of the world's population [1,2]. It is caused by *Toxoplasma gondii* (*T. gondii*), an obligate intracellular protozoan parasite [3]. This parasite, whose definitive host is the cat or other felids, can affect all warm-blooded animals, including humans [4]. Toxoplasmosis prevalence is less than 10% in hot and dry climates and between 60 and 80% in humid areas [5]. It is generally higher in the Americas and sub-Saharan Africa due to the abundance of cats and a climate favorable to oocysts survival [6]. Toxoplasmosis is asymptomatic in immunocompetent individuals, but is serious in immunocompromised individuals and pregnant women. In the latter, the absence of immunity to *T. gondii* exposes the individual to primary infection and the risk of foetal transmission, which can lead to early abortion and congenital malformations [7,8]. The risk of foetal infection increases with gestational age at the time of infection, and is often highest in the third trimester. Foetal damage is more severe when maternal infection occurs in the first trimester [9]. It is therefore of great interest to monitor the immunological status of toxoplasmosis in pregnant women. In the latter, immunological monitoring is based on the detection of markers of infection, notably IgG and IgM antibodies to *T. gondii*. One week after the onset of infection, IgM, a marker of recent infection, is the first to appear; and IgG, a marker of old infection, appears 2 to 3 weeks after IgM. Depending on the pattern presented, infection is considered acute (IgG+/IgM+), recent (IgG-/IgM+), chronic (IgG+/IgM-), and absent (IgG-/IgM-). [10,11]. In Cameroon, confirmation of the first two profiles leads to drug treatment. Thus, *T gondii* is investigated in pregnant women using serological techniques. However, the use of these tools remains a concern. Indeed, the techniques are numerous; they differ both in their principles and in the nature of the reagents used; also there are even differences in the interpretation of the results [12,13]. In Cameroon, some studies have shown that the prevalence of toxoplasmosis in pregnant women varies from one year to another, from one region to another, and from one city to another. Thus, from 1981, it was 78.6% in Douala [14], 65% in Penka-Michel [15], and 35.77% in Buea, [16]. The serological diagnostic techniques applied in local studies have been diverse: Enzyme Immuno Assay (EIA) [15], RDT [17], and ELISA [18]. In the absence of national planning, do current strategies for diagnosing toxoplasmosis in pregnant women improve management in Cameroon? In order to contribute to the improvement of the management of toxoplasmosis in pregnant women in Cameroon, we evaluated the performance of two techniques immunochromatographic and immunoenzymatic commonly used in health facilities.

## Methods

A multicenter, cross-sectional, descriptive and analytical study was conducted from June 2019 to February 2020 in seven Catholic health facilities in the Center (03) and Littoral (04) regions. The health facilities in the Center were located in urban areas. Those in the Littoral were equally divided between urban and rural areas. In the health facilities using the ICT method (by RDT), the pregnant women recruited were grouped in batch 1 (341), and those using the IEZ method (by ELISA) in batch 2 (200).

**Inclusion criteria:** the study population consisted of pregnant women aged 14-46 years who were seen for the first time at antenatal clinics (ANC) in the health facilities selected for the study.

**Sample size:** based on the average of the toxoplasmosis prevalence values published by Njunda *et al*. [19] in the Center i.e. 65.5% and Nguefack *et al*. [14] in the Littoral i.e. 78.6%, a minimum sample size of 310 participants was obtained from the formula:


$$n=\frac{z^{2}\times P\times \left(1-P\right)}{d^{2}}\;\left[20\right].$$

In this formula, n = sample size, Z (1.96) = the statistic corresponding to level of confidence with 95%, P = expected prevalence and d = precision´s level at 0.05.

**Sample analysis:** for each participant, socio-demographic data were collected on individual survey sheets. Next, 3mL x 2 of venous blood were collected and divided into two dry tubes. The first tube was used for serological analysis (RDT or ELISA) in the health facilities. The description of the kits used is given in Annex 1. The second tube of blood was sent to the reference laboratory: the Immunology and Microbiology Laboratory (LIM) of the Chantal BIYA International Reference Center (CIRCB). In the laboratory, the tube was left to rest for 1 hour. After coagulation, it was centrifuged at 3000 rpm for 15 minutes; the serum obtained was aliquoted in cryotubes and stored at -80°C. Anti-toxoplasma IgG and IgM antibodies were tested by ICT (TDR_LIM_), EIA (ELISA_LIM_), and ELFA methods of VIDAS Biomérieux the latter being taken as reference method. In the health facilities included in this study, the serological diagnosis of toxoplasmosis in pregnant women is carried out by the ICT or IEZ techniques. As the harmonization of these methods is not effective in our context, we choose the ELFA method from VIDAS Biomérieux as a reference. This is an automated test based on a two-step IEZ assay for IgG and immunocapture for IgM with a final detection by fluorescence [21-23]. The description of the kits used at the LIM is given in Annex 2.

**Data analysis:** the categorical variables are presented as numbers and frequencies. Chi-square and Fisher's exact tests were used to compare the proportions of the different categorical variables using SPSS version 25 software (SPSS, Inc., Chicago, IL, USA). The Z value (Z) was calculated for the comparison of the two methods with matched data. Sensitivity (Se) and specificity (Sp) were calculated using the software [24]. The Youden index and the kappa agreement coefficient (Ka) were also calculated. The significance level for differences was set at p < 0.05.

**Ethical considerations:** the protocol of this study received the approval of the National Ethics Committee through the issuance of the Ethical Clearance N°2019/05/18/CE/CNERSH/SP, as well as the administrative authorisations from the managers of the different health facilities retained. Written informed consent was also obtained from each eligible and willing pregnant woman.

## Results

**Sociodemographic profile of pregnant women:** the project was presented to 1070 pregnant women, 50.6% (541) consented to participate. Their ages ranged from 14 to 46 years, with an average of 27.67 ± 5.58 years. The modal class was (21-31) years representing 61.4% (332/541) of the study sample; 63.2% were unmarried; 58.4% had attained secondary education; 57.9% were registered in the 2nd trimester of pregnancy; 72.2% were in at least their second pregnancy; 73.9% had no knowledge of toxoplasmosis; 64% did not recognize the presence of a cat in their environment.

**Toxoplasmosis infection rates obtained in the health facilities with RDT:** only 13.3% of samples were positive. With the VIDAS ELFA method, the infection rate was 6 times higher (80.4%). The difference between the two methods was significant between the infection rates of IgG (p<0.0001) (Table 1).

**Table 1:** infection rates of anti-toxoplasma IgG in RDT facilities

Batch 1 N=341		IgG/ELFA n (%)			Batch 1 N=341		IgG/RDT n (%)		
**IgM / ELFA**		Positive	Negative	Total	**IgM/RDT**		Positive	Negative	Total
	Positive	3 (0.9)	0 (0)	3 (0.9)		positive	21 (6.2)	6 (1.8)	27 (8)
	Negative	271 (79.5)	67 (19.6)	338 (99.1)		negative	18 (5.2)	296 (86.8)	314 (92)
	Total	274 (80.4)	67 (19.6)	341 (100)		Total	39 (11.4)	302 (88.6)	341 (100)
				%					%
Prevalence of toxoplasmosis by ELFA				80.4	Prevalence of toxoplasmosis by RDT_Batch1_				13.2
IgG+/IgM+ (acute infection)				0.9	IgG+/IgM+ (acute infection)				6.2
IgG+/IgM- (chronic/past infection)				79.5	IgG+/IgM- (chronic/past infection)				5.2
IgG-/IgM+ (progressive infection/IgM fugitive)				0	IgG-/IgM+ (progressive infection/IgM fugitive)				1.8
IgG-/IgM- (no infection))				19.6	IgG-/IgM- (no infection))				86.8
IgG-rate				80.4	IgG-rate				11.4
IgM-rate				0.9	IgM-rate				8

Batch 1=groups of hospitals under RDT, ELFA=enzyme-linked fluorescent assay, Ig=immunoglobulin, N=sample size, n (%)=number and percentage, RDT=rapid diagnostic test For IgG, Z1 = 15 with p <0.0001 For IgM, Z2 = 15 with p <0.0001,

**Toxoplasmosis infection rates obtained in health facilities using ELISA:** the infection rates of toxoplasmosis obtained by ELISA Batch2 and ELFA were similar at 75% and 74% respectively. For IgG, the difference between the infection rates determined by these two methods was not significant (p>0.05), but was significant for IgM (p<0.05). (Table 2).

**Table 2:** frequencies of anti-toxoplasma IgG and IgM in health facilities under ELISA

Batch 2 N=200		IgG / ELFA n (%)			Batch 2 N=200		IgG/ELISA n (%)		
IgM/ELFA		Positive	Negative	Total	IgM/ELISA		Positive	Negative	Total
	Positive	7 (3.5)	1 (0.5)	8 (4)		positive	20 (10)	0 (0)	20 (10)
	negative	140 (70)	52 (26)	192 (96)		negative	130 (65)	50 (25)	180 (90)
	Total	147 (73.5)	53(26.5)	200 (100)		Total	150 (75)	50 (25)	200 (100)
				%					%
Prevalence of toxoplasmosis by ELFA				74	Prevalence of toxoplasmosis by RDT_Batch2_				75
IgG+/IgM+ (acute infection)				3.5	IgG+/IgM+ (acute infection)				10
IgG+/IgM- (chronic/past infection)				70	IgG+/IgM- (chronic/past infection)				65
IgG-/IgM+ (progressive infection/IgM fugitive)				0.5	IgG-/IgM+ (Progressive infection/IgM fugitive)				0
IgG-/IgM- (no infection)				26	IgG-/IgM- (no infection))				25
IgG-rate				73.5	IgG-rate				75
IgM-rate				4	IgM-rate				10

Batch 2=groups of hospitals under ELISA, ELFA=enzyme-linked fluorescent assay, ELISA=enzyme linked immunosorbent assay, Ig=immunoglobulin, N=sample size, n (%)=number and percentage For IgG, Z1 = 15 with p <0.0001 For IgM, Z2 = 15 with p <0.0001

**Toxoplasmosis infection rates obtained in the Immunology and Microbiology Laboratory of the CIRCB on the two batches of samples:** in the subpopulation 1, the toxoplasmosis infection rate was 76.8% with TDR_LIM_ and 86.8% with ELISA_LIM_. In subpopulation 2, these rates ranged from 71% with TDR_LIM_ to 81.5% with ELISA_LIM_. For both anti-*T. gondii* antibody assays, the IgG levels obtained by the two methods were different in the two batches (p < 0.0001). On the other hand, the IgM frequencies obtained by the two methods were statistically similar in Batch 1 (p=0.32), and for Batch 2 (p=0.65) (Table 3).

**Table 3:** toxoplasmosis infection rates obtained in the laboratory with the RDT and ELISA on the two batches

Batch	Experienced technique	IgG+n (%)	Z (P-value)	IgM+ n (%)	Z (P-value)
N1 =341	RDT_LIM_	262 (76.8)	5.33 (<0.0001)	2 (0.6)	1 (0.32)
	ELISA_LIM_	294 (86.2)		1 (0.3)	
N2 = 200	RDT_LIM_	142 (71)	4.26 (<0.0001)	3 (1.5)	0.45 (0.65)
	ELISA_LIM_	162 (81)		4 (2)	

**Batch**=groups of hospitals**, ELISA**=Enzyme Linked Immunosorbent Assay, **ELISA_LIM_ =** ELISA at LIM, **Ig**=Immunoglobulin**, N1**=sample size in hospitals under RDT, **N2**=sample size in hospitals under ELISA, **n (%)=**number and percentage, **ELISA**=enzyme linked immunosorbent assay, **RDT=**rapid diagnostic test, **RDT_LIM_ =**RDT at LIM, **Z=**Z value

**Performance of immunochromatographic and enzyme immunoassay methods**

**IgG detection:** for the detection of IgG in the first batch of samples, the RDT_Batch1_ applied in the health facilities was not very efficient and less concordant with the reference, it was highly specific and poorly sensitive. Its positive predictive value (PPV) was above 80%, and the negative predictive value (NPV) very low (20%). On the other hand, the two methods tested at LIM on the same samples showed similar performances; they were very sensitive but less specific. Their predictive values were similar, over 90% for PPV, and over 70% for NPV. The values of the Youden index (0.6) and the kappa coefficient (≥ 0.7) indicate that both experimental methods are much better (Table 4). However, in the second batch, the ELISA technique applied in the hospitals and our two experimental methods showed similar and good performance in the IgG assay. For all three techniques, the sensitivity was > 90%, the specificity > 70%. In this second batch, PPV and NPV were above 80%. Furthermore, the Youden index values (≥ 0.7) indicate that all three methods are good and well suited for IgG detection (Table 4).

**Table 4:** performance of the RDT and ELISA for the detection of IgG and IgM

	Batch	Performance	RDT_Hop_	RDT_LIM_	ELISA_LIM_
IgG [% (CI à 95%)]	1	Sensitivity	12 (8.4-16.5)	96 (92.9-98)	97.5 (94.8-99)
		Specificity	91 (81.5-96.6)	64.2 (51.5-75.5)	59.7 (47-71.5)
		PPV	84.6 (70.6-92.6)	91.6 (88.8-93.8)	90.8 (88.1-93)
		NPP	20.20 (18.8-21.6)	79.6 (68.1-87.8)	85.1 (72.8-92.4)
		Youden Index	0.03	0.6	0.6
		Kappa	0.01	0.8	0.7
	2		**ELISA_Hop_**	**RDT_LIM_**	**ELISA_LIM_**
		Sensitivity	95.2 (90.4-98.1)	94.6 (89.6-97.6)	99.3 (96.3-99.98)
		Specificity	75.5 (61.7-86.2)	96.2 (87-99.5)	70 (71.4-93)
		PPV	91.5 (87.0-94.5)	98.6 (94.7-99.6)	90.1 (85.8-93.2)
		NPP	85.1 (73.2-92.3)	86.4 (76.4-92.6)	97.3 (83.7)-99.6)
		Youden index	0.7	0.9	0.7
		Kappa	0.8	0.8	0.7
**IgM** [% (CI à 95%)]	1		**TDR_Hop_**	**RDT_LIM_**	**ELISA_LIM_**
		Sensitivity	33.3 (0.8-90.6)	33.3 (0.8-90.6)	33.3 (0.8-90.6)
		Specificity	92.3 (88.9-94.9)	99.7 (98.4-99.9)	100 (98.91-100)
		PPV	3.7 (0.7-16.6)	50.0 (7.4-92.6)	100
		NPP	99.4 (98.6-99.7)	99.4 (98.7-99.7)	99.4 (98.7-99.7)
		Youden index	0.3	0.3	0.3
		Kappa	0.1	0.2	0.2
	2		**ELISA_Hop_**	**RDT_LIM_**	**ELISA_LIM_**
		Sensitivity	37.5 (8.5-75.5)	25 (3.2-65.1)	12.5 (0.3-52.7)
		Specificity	91.2 (86.2-94.8)	97 (93.3-98.8)	98.4 (95.5-99.7)
		PPV	15.0 (6.1-32.5)	25.0 (7.4-58.4)	25.0 (7.4-58.4)
		NPP	97.2 (95.3-98.4)	96.9 (95.4-97.9)	96.4 (95.4-97.2)
		Youden Index	0.3	0.2	0.1
		Kappa	0.2	0.2	0.2

**Batch =** Group of Hospitals. **CI=**Confidence interval. **Batch 1**=groups of hospitals under RDT. Batch 2=groups of hospitals under ELISA**. ELISA_Hosp_**=ELISA tested in Batch2. **ELISA_LIM_**=ELISA of LIM. **%**=percentage. **PPV**=Positives predictive value. **NPV**= Negative predictive value. **RDT=**Rapid diagnostic test**. RDT_Hosp_**= RDT tested in Batch1. **RDTLIM** =RDT of LIM.

**IgM detection:** for the IgM assay, the methods used both in the health facilities and in the Immunology and Microbiology Laboratory of the CIRCB were specific (Sp > 91%) but not very sensitive (Se < 38%). For the IgM assay, the different methods presented in both batches showed NPVs above 90%. In contrast, the PPVs were below 30% for the RDT_Batch1_ in batch 1 and in all 3 methods in batch 2. All of them also appeared to be not very effective, since the Youden index ≤ 0.3 (Table 4).

## Discussion

In order to contribute to the improvement of the management of toxoplasmosis in pregnant women in Cameroon, we determined the prevalence of toxoplasmosis with two techniques routinely used in hospitals; we further evaluated the performance of these techniques. This is the first multicenter study of its kind in the diagnosis of toxoplasmosis in Cameroon. Two groups of serological test results collected respectively from health facilities (RDT_Batch1_ and ELISA_Batch2_) and at the Immunology and Microbiology Laboratory (LIM) of the CIRCB (RDT_LIM_ and ELISA_LIM_) are presented. The participants were recruited from 7 health facilities; 5 of which were tested with RDT and 2 with ELISA for biological diagnosis of toxoplasmosis. Of these pregnant women, 341 were recruited in five health facilities (Batch 1) and 200 in the other two (Batch 2). In Batch1, the prevalence of toxoplasmosis in pregnant women using the ICT/RDT method was 13.3%. This rate is much lower than those reported by previous studies in pregnant women in Cameroon using the same technics, i.e. 22.84% in Yaoundé [25], 35.4% in Bamenda [26], 22.9% in Buea [16], and 32.4% in Yaoundé [27]. Our prevalence (13.3%) is also lower than other studies conducted with the ICT method in West Africa, namely 44.4% in Nigeria [28] and 48.5% in Benin [29]. At LIM/CIRCB, the ICT and EIA methods applied to the same samples gave infection rates of 76.8% and 86.8% respectively, well above 13.3%. This discrepancy could be related to management of inventories, storage procedures, validity of the tests at the time of use, and the competence of the laboratory staff. Several cases of toxoplasmosis would therefore be ignored, which would give rise to risks of neonatal infections, premature abortions, etc.

Using the IEZ/ELISA method, the seroprevalence of toxoplasmosis in Batch 2 was 75%. This result is close to the data from other studies carried out in pregnant women using the same method, namely 70% and 78.6% in the city of Douala [14,30] in Cameroon. It is also close to 71% obtained by the ICT method tested at the LIM of CIRCB. Nevertheless, this result (75%) collected from health facilities is slightly lower than 81.4%, the infection rate reported in Ethiopia [31], and 81% obtained at LIM with the IEZ technique. However, this seroprevalence (75%) is higher than the 36.5% obtained in Mbouo-Mbandjoun (West Region of Cameroon) [18] and 65.5% obtained in Yaoundé [19] determined by the same technics. Moreover, it is also higher than that of studies conducted outside Cameroon using the same method in pregnant women, i.e. 69% in Brazil [32] and 48.9% in Nigeria [6]. The locality or environment, cultural dietary habits, thus seem to have an impact on the probability of contacting toxoplasmosis [5].

The results collected from the health facilities and those obtained at LIM/CIRCB show differences in the prevalence of anti-toxoplasmic IgG/IgM. In the health facilities as well as in the Immunology and Microbiology Laboratory of the CIRCB, for the ELISA technique, the number of sera containing IgG was higher than with the RDT. However, both methods were equivalent in the detection of IgM. The differences observed between the infection rates reported by the health facilities in our study and those obtained at the LIM of the CIRCB, using the same methods and on the same samples, illustrate the difficulties in interpreting serological results for toxoplasmosis [13]. In Batch1, the sensitivity of the RDT (12%) for IgG detection is much lower than the 54.4% obtained in Egypt [33], the 46.7% found in Nigeria [34], and the 96% obtained at LIM/CIRCB with the same technique. These differences could be related to the handling conditions on the one hand and the quality of the kits used on the other. The value of Se/RDT_Batch1_=12% is also lower than 97% obtained in Benin [29]. This other difference in sensitivity would be related to the difference in the principles of the methods applied [35]; indeed, the RDT applied in the Benin study was based on an enzyme-linked immunosorbent method, which could justify its best performance. This is supported by the high sensitivity level of 97.45% obtained at LIM with the ELISA technique tested on the same batch of samples (Table 4). In subpopulation 2, the ELISA_Batch2_ technique applied in health facilities showed similar performance to our two experimental methods against IgG (Se ≥ 90% and Sp ≥ 70%). A study carried out in Nantes (France) showed comparable performance (Se=98.87% and Sp=88.46%) when the authors compared the Platelia microplate technique based on the Elisa principle and the Vidas Biomérieux automated system [36].

Regarding IgM detection, our two experimental techniques and those applied to the two batches of health facilities showed equivalent performance. Their sensitivity was 33.3%. This result is close to 33.3% and 29.03%, sensitivities obtained respectively in Benin [29] and Egypt [33]. The sensitivity of the TDR_Batch1_ towards IgM therefore still appears to be low in this case. This could be partly due to the large number of false negatives recorded. This low sensitivity of the TDR_Batch1_ to detect IgM could pose a problem in the diagnosis of acute toxoplasma infections in pregnant women; these results suggest the use of a confirmatory test for IgM [37,38]. In the second batch of samples, the ELISA_Batch2_ technique used in hospital centres and the two techniques tested at the LIM had also very low sensitivity (Se ≤ 30%) although quite specificity (Sp ≥ 90%). This result differs from that of Martin & Morin [36] who found a sensitivity of 87.08% and a specificity of 96.97% with the same technique. The low sensitivity of IgM recorded in the current study is already reflected in the low positivity rate (10%) (Table 2). This could be explained by the fact that the majority of women recruited in this study were highly immunized on the one hand, RDT and ELISA tests applied both in the hospitals retained in this study and at the LIM of the CIRCB might not be sufficiently sensitive to detect acute infections on the other hand.

**Limitations:** the bias of this study is that the two groups of results were not obtained on identical samples. The non-uniformity of the numbers in the different health facilities did not allow a good comparison by sampling site and by kit.

## Conclusion

The infection rate of toxoplasmosis among pregnant women in health facilities that used RDT_Batch1_ was 13.2%; and 75% for those that used ELISA_Batch2_. In the laboratory, the prevalences obtained in this study were above 70% for RDT_LIM_ and ELISA_LIM_. Both experimental techniques showed similar performance (good for IgG and poor for IgM). Moreover, only the ELISA_Batch2_ technique showed similar performance to our experiments. The differences in performances observed between the RDTLIM and the one applied in the health facilities suggest that evaluations should be extended with a view of harmonizing diagnostic technique for toxoplasmosis in pregnant women in Cameroonian health facilities.

### What is known about this topic

- *Toxoplasmosis is a public health problem with severe consequences in pregnant women;*
- *The use of a variety of serological assays remains a concern and presents a real difficulty to interpret the results, especially in countries with limited resources*.

### What this study adds

- *For IgG detection, the ICT method used in the health facilities was less efficient than the one we tested, with good performance (sensitive and specific); on the other hand, the IEZ method applied in the health facilities and the one used in our experiments showed similar performance (sensitive and specific) in the IgG assay;*
- *Both methods (ICT and IEZ) were less sensitive and less efficient for IgM detection;*
- *There is a need for harmonisation of serological diagnostic methods in health facilities in Cameroon*.

## Annexes

**Annex 1**: description of the kits used in the health facilities (319 KB)

**Annex 2**: description of the kits used at the CIRCB Laboratory (320 KB)
